# Sex Differences in the Association Between Ultra-Processed Food Consumption and NAFLD: An Analysis of KNHANES 2013–2021 Data

**DOI:** 10.3390/jcm14227930

**Published:** 2025-11-08

**Authors:** Byung Soo Kwan, Nak Gyeong Ko, Ji Eun Park

**Affiliations:** 1 Department of Internal Medicine, Samsung Changwon Hospital, Sungkyunkwan University School of Medicine, Changwon 51353, Republic of Korea; kbs2459@naver.com; 2 Department of Research & Support, Samsung Changwon Hospital, Sungkyunkwan University School of Medicine, Changwon 51353, Republic of Korea; 2022230@smc.skku.edu; 3 Department of Obstetrics and Gynecology, Gyeongsang National University Changwon Hospital, Changwon 51472, Republic of Korea; 4 Department of Obstetrics and Gynecology, Gyeongsang National University, Jinju 52727, Republic of Korea; 5 Institute of Medical Science, Gyeongsang National University, Jinju 52727, Republic of Korea

**Keywords:** diet, nonalcoholic fatty liver disease, ultra-processed foods

## Abstract

**Background/Objectives:** Ultra-processed food (UPF) consumption is increasingly implicated in metabolic diseases; however, evidence for non-alcoholic fatty liver disease (NAFLD) and potential sex differences remains limited. Thus, this study aimed to examine the relationship between UPF consumption and NAFLD stratified by sex in Korean adults. **Methods**: This was a cross-sectional analysis of Korean adults from the Korea National Health and Nutrition Examination Survey 2013–2021 (*n* = 24,587). UPF intake was quantified as the percentage of NOVA Group 4 items consumed in total daily food weight based on a 24 h recall. The participants were grouped into quartiles of UPF intake. NAFLD was defined using the hepatic steatosis index. Survey-weighted models were used to summarize the characteristics and estimated odds ratios (ORs) for NAFLD across the UPF quartiles with adjustment for factors associated with both NAFLD and dietary intake. Linear trend tests across quartiles and continuous analyses of UPF intake were performed, with sex-stratified models to assess potential effect modification. **Results**: NAFLD prevalence increased as UPF intake quartile increased, from 19.1% in Q1 to 24.1% in Q4. With Q1 as reference, the fully adjusted OR for Q4 was 1.24 (95% CI, 1.10–1.41, *p* for trend = 0.001). In the sex-stratified analyses, the association was only significant in women (Q4 vs. Q1: OR, 1.52, 95% CI, 1.28–1.81; *p* for trend < 0.001). When UPF intake was modeled as a continuous variable, NAFLD risk showed a modest overall increase, with a nearly flat pattern in men and a clear linear increase in women. **Conclusions**: Higher UPF intake is associated with a greater risk of NAFLD in Korean adults, with a more pronounced association in women. Thus, UPF consumption is a feasible modifiable target for liver health.

## 1. Introduction

Non-alcoholic fatty liver disease (NAFLD) is defined as excessive hepatic fat accumulation in the absence of significant alcohol intake. Importantly, NAFLD is the most common chronic liver disorder worldwide, prevalent in approximately more than 30% of adults [1] and especially more prevalent in individuals with obesity or type 2 diabetes mellitus than in the general population [2]. In recognition of its strong metabolic basis, the terminology for non-alcoholic fatty liver disease (NAFLD) has evolved. The term metabolic-associated fatty liver disease (MAFLD) was previously proposed to better capture the metabolic nature of the disease, and more recently, a multisociety Delphi consensus has endorsed the term metabolic dysfunction-associated steatotic liver disease (MASLD), providing a non-stigmatizing and inclusive nomenclature that highlights metabolic dysfunction as the key driver of disease pathogenesis [3]. NAFLD can progress from simple steatosis to non-alcoholic steatohepatitis and hepatic fibrosis and is associated with an elevated risk of cardiovascular disease and other complications. Alarmingly, young adults with NAFLD have a five-fold higher risk of mortality than those without the condition [4].

Ultra-processed foods (UPFs), classified as Group 4 in the NOVA food classification system [5], are industrial formulations produced through extensive mechanical and chemical processing, often incorporating additives to enhance shelf life, flavor, and texture. Over the past several decades, global UPF consumption has risen to strikingly high levels; in the United States, these products now provide approximately 53–58% of total daily energy intake among adults [6], with similar or higher proportions reported in other high-income countries. In some Western populations, they constitute more than half of all calories consumed [7]. This increasing dietary share has drawn attention to its potential health effects. High UPF consumption has been consistently linked to obesity, dyslipidemia, hypertension, cardiovascular disease, type 2 diabetes, and certain cancers [8]. Cohort studies have further demonstrated higher rates of metabolic syndrome and all-cause mortality in individuals with greater UPF intake than in those with lower UPF intake [9,10].

Genetic predisposition and lifestyle factors, particularly diet, are central to NAFLD pathogenesis. Emerging evidence suggests that higher UPF consumption is associated with an increased risk of NAFLD. A recent meta-analysis estimated a 22% higher risk of NAFLD among individuals in the highest UPF intake category than those among in the lowest [11]. However, substantial between-study heterogeneity was observed (I^2^ = 78.5%), likely reflecting differences in study design, participant characteristics, food classification methods, and NAFLD diagnostic criteria. In addition, a hospital-based cross-sectional study from Israel found no significant association between UPF intake and NAFLD [12]. These inconsistencies highlight the need for studies that account for population-specific dietary patterns and metabolic susceptibilities.

Racial and ethnic disparities in the prevalence of NAFLD have also been reported [13,14]. However, most prior evidence has originated from Western populations, and data from Asian cohorts are scarce. Cultural and genetic differences may influence both UPF consumption patterns and metabolic responses to such diets, limiting the generalizability of Western findings to Asian populations. Moreover, NAFLD prevalence and risk factors differ according to sex [15], suggesting that sex-stratified analyses can reveal distinct associations between UPF intake and NAFLD.

This study aimed to examine the relationship between UPF consumption and NAFLD stratified by sex in a nationally representative sample of Korean adults using 2013–2021 Korea National Health and Nutrition Examination Survey (KNHANES) data. Toward this goal, a sex-stratified analyses of the association between NALFD and UPF consumption, determined from 24 h dietary recall data, were performed to assess potential effect modifications.

## 2. Materials and Methods

### 2.1. Study Design and Population

**Figure 1:**
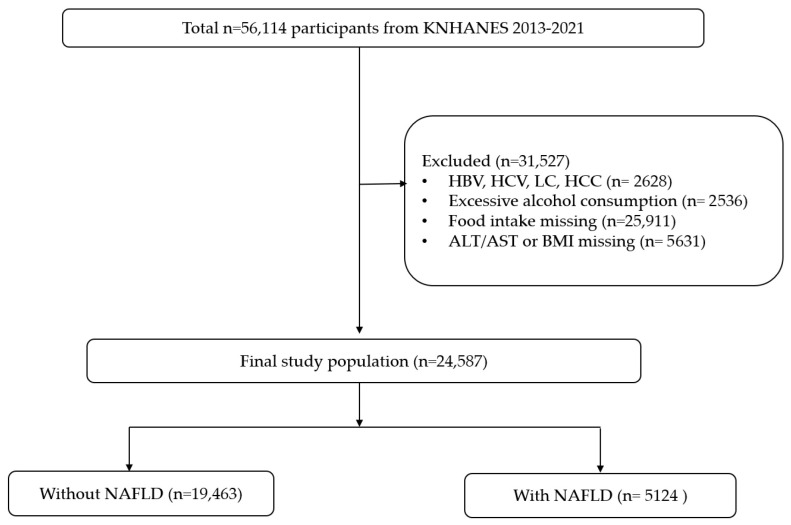
Study flow diagram.

This cross-sectional analysis was conducted using data from the 2013–2021 KNHANES. Briefly, the KNHANES is an annual nationwide survey that uses a stratified multistage probability sampling design to obtain a representative sample of the non-institutionalized Korean population. The study population included adults aged ≥19 years who participated in the health examination and nutrition survey. Participants with known chronic liver conditions (i.e., with positive hepatitis B or C markers, history of liver cirrhosis or liver cancer) or significant alcohol use (i.e., with evidence of high-risk alcohol consumption) were excluded. Individuals with missing outcome or dietary data were also excluded. In total, 24,587 adults were included in the analysis (Figure 1).

### 2.2. Exposure Assessment: Ultra-Processed Food Intake

Dietary intake was assessed using a single 24 h recall interview conducted by trained dietitians as part of the KNHANES. Each reported food item was classified according to the NOVA food processing classification system [5]. This system categorizes foods into four groups according to the degree of processing, with Group 4 representing UPFs. Given the distinct characteristics of the Korean diet, the standard NOVA framework was further specified to reflect local dietary patterns and ensure the accurate classification of traditional and composite dishes [16]. Two trained researchers independently reviewed and coded all reported foods into NOVA categories; disagreements were resolved by consensus.

UPF intake for each participant was quantified as a percentage of the total daily food weight of NOVA Group 4 items consumed. This percentage was calculated by dividing the total weight (g) of all UPF items consumed in the 24 h recall by the participant’s total daily food intake (g) and multiplying by 100. The participants were then divided according to the quartiles of the calculated percentages, with the lowest quartile (Q1) used as the reference group for all analyses.

### 2.3. Outcome Definition: NAFLD

The outcome of interest was NAFLD, defined using the HSI, a validated noninvasive marker of fatty liver [17]. The HSI for each participant was calculated as 8 × (serum alanine aminotransferase/serum aspartate aminotransferase levels) + BMI. Consistent with literature, presumed NAFLD was determined as an HSI value exceeding sex-specific thresholds of >36 in men and >34 in women.

### 2.4. Covariates

The analysis was adjusted for demographic, socioeconomic, lifestyle, anthropometric, and dietary factors with established associations with both NAFLD and dietary intake. Demographic and socioeconomic variables included age (years), sex (male or female), educational attainment (elementary school or lower, middle school, high school, or college and above), and household income level (quartiles from lowest to highest). Lifestyle variables included smoking status (current smoker or nonsmoker), alcohol consumption frequency (less than once per month or at least once per month), and physical activity (meeting or not meeting the national aerobic exercise guidelines [18]). Physical activity in the KNHANES was defined based on aerobic activity only, engaging in ≥150 min per week of moderate-intensity or ≥75 min per week of vigorous-intensity aerobic activity, or an equivalent combination of both. Resistance training was not included in this definition. BMI (kg/m^2^) was included as a measure of adiposity in the baseline characteristics but was not treated as a covariate in the regression models, as BMI is already incorporated into the hepatic steatosis index (HSI) used to define NAFLD. Total daily energy intake (kilocalories) from the 24 h dietary recall was included to account for overall caloric consumption. All covariates were derived from the standardized KNHANES health examination, health interview, and nutrition survey components and were selected a priori based on their known relevance to NAFLD risk and diet quality.

### 2.5. Statistical Analysis

The analyses were conducted in two steps. First, the baseline characteristics of the participants were compared across quartiles of UPF intake. Continuous variables were expressed as the mean ± standard deviation and categorical variables as number (percentage). Group differences were evaluated using analysis of variance for continuous variables and Pearson’s chi-square test for categorical variables. Second, the association between UPF intake and NAFLD was assessed using multivariate logistic regression. Odds ratios (ORs) and 95% confidence intervals (CIs) were estimated for each quartile (Q2–Q4) with Q1 as the reference after adjusting for prespecified covariates (age, sex, education, income, smoking, alcohol, physical activity, and total energy intake). Furthermore, UPF intake was modeled as a continuous variable to evaluate the dose–response relationship with NAFLD. Multivariable logistic regression was performed using the percentage of total food intake from UPF as a continuous predictor. Predicted odds and 95% confidence intervals were estimated across the continuous distribution of UPF intake and presented graphically with a fitted regression line and 95% CI bands. Linear trends were tested by modeling UPF intake as a continuous variable in logistic regression models. Sex-stratified analyses were also performed to examine potential effect modifications. All analyses accounted for the complex sampling design of the KNHANES, incorporating sampling weights to ensure national representativeness. All statistical analyses were performed using Stata 15.1 (Stata Corporation, College Station, TX, USA) and R 4.4.2 (Vienna, Austria; http://www.R-project.org/ (accessed on 22 August 2025)). A two-sided *p* value of <0.05 was considered significant.

## 3. Results

### 3.1. Characteristics of the Study Population

UPFs accounted for a median of 12.85% (interquartile range [IQR], 4.96–26.58%) in total foods consumed. The cut-off values for UPF intake quartiles were <4.96% for Q1; 4.96–9.96%, Q2; >9.96–26.58%, Q3; and >26.58%, Q4 (Table 1). In total, 6147; 6146; 6148; and 6146 participants belonged to the Q1, Q2, Q3, and Q4 groups, respectively. The participants in the higher UPF quartile groups tended to be younger (mean age: 38 years in the Q4 group vs. 48 years in the Q1 group) and were more often male (60% in the Q4 group vs. 39% in the Q1 group). The proportion of participants with college-level education or higher was greater whereas the proportion of participants with elementary school education or lower was lower in the Q4 group. Household income levels were slightly higher in the upper quartiles. Lifestyle patterns also differed; the proportion of current drinkers increased from 49% in the Q1 group to 75% in the Q4 group and the proportion of current smokers from 11% to 29%. Meanwhile, the proportion of participants engaging in regular physical activity decreased from 53% in the Q4 group to 48% in the Q1 group. The mean total energy intake increased from 1940 kcal/day in the Q1 group to 2385 kcal/day in the Q4 group.

### 3.2. Association Between UPF Intake and NAFLD

The unadjusted and multivariate-adjusted logistic regression results for the association between %UPF intake quartiles and NAFLD, with Q1 as the reference category, are presented in Table 2. The prevalence of NAFLD increased from 19.1% in the Q1 group to 24.1% in the Q4 group. In the unadjusted logistic regression, Q4 was associated with a higher odds of NAFLD compared with Q1 (OR, 1.34; 95% CI, 1.21–1.49; *p* for trend <0.001; Table 2). The association remained significant after adjusting for age and sex (OR, 1.16; 95% CI, 1.04–1.30). In the fully adjusted model controlled for age, sex, education level, household income, smoking status, alcohol consumption, physical activity, and total energy intake, the risk of NAFLD was 24% higher in Q4 than in Q1 (OR, 1.24; 95% CI, 1.10–1.41; *p* for trend = 0.001).

### 3.3. Sex-Stratified Analyses

Among men, NAFLD was prevalent in 25% and 28% of the participants in the Q1 and Q4 groups, respectively. There was no significant association between Q4 and Q1 in the fully adjusted analysis (OR, 1.06; 95% CI, 0.89–1.26; *p* for trend = 0.943). Among women, NAFLD was prevalent in 15.3% in Q1 and 17.1% in Q4. In the fully adjusted model, the odds of NAFLD was significantly higher in women in the Q4 group than in those in the Q1 group (OR, 1.52; 95% CI, 1.28–1.81; *p* for trend <0.001). The multivariate logistic regression estimates for NAFLD prevalence by %UPF consumption quartiles in the fully adjusted model are presented in Table 2.

### 3.4. Continuous Trends in NAFLD Risk Across UPF Intake

**Figure 2:**
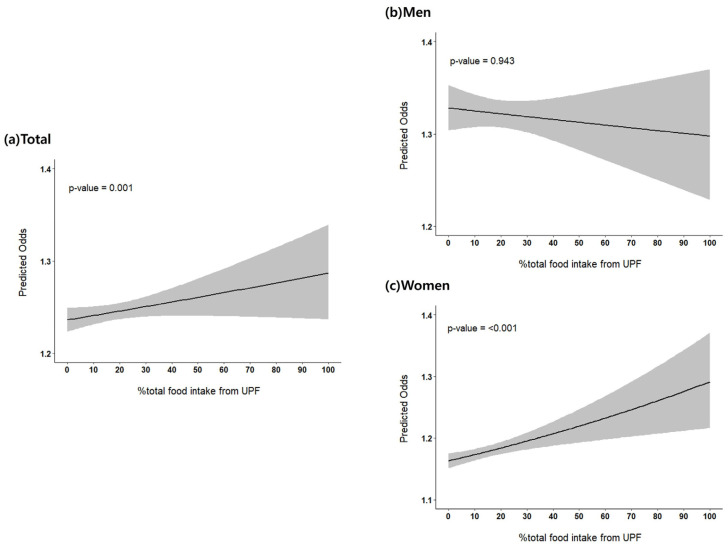
Dose–response relationship between UPF intake and NAFLD. Multivariate logistic regression models were used to estimate predicted odds (95% confidence intervals [CIs]) for NAFLD across the continuous distribution of %UPF consumption in the (**a**) total, (**b**) men, and (**c**) women populations. Solid lines represent point estimates and shaded areas indicate 95% CIs. The models are adjusted for age, education, income, smoking, alcohol, physical activity, and total energy intake.

When UPF intake was modeled as a continuous variable, a significant positive association with NAFLD was observed in the overall population (Figure 2a). The predicted odds of NAFLD increased progressively with higher UPF consumption, and the linear trend was statistically significant (*p*-value = 0.001). In sex-stratified analyses, no significant association was found in men (Figure 2b), with a nearly flat slope and a non-significant linear trend (*p* for trend = 0.943). In contrast, women showed a clear dose–response pattern (Figure 2c), with steadily increasing predicted odds of NAFLD across the continuum of UPF intake, and the linear trend was highly significant (*p*-value < 0.001).

## 4. Discussion

In this analysis of a large, nationally representative cohort of Korean adults spanning 2013 to 2021, greater consumption of UPFs was significantly associated with a higher prevalence of NAFLD. Compared with those in the lowest quartile, participants in the highest quartile of UPF intake had approximately 24% higher odds of NAFLD, even after adjusting for demographic, socioeconomic, and lifestyle factors. A clear dose–response relationship was observed, with NAFLD prevalence increasing from approximately 19% in the lowest UPF quartile group to 24% in the highest UPF quartile group. Importantly, this association differed by sex: while there was no significant association after multivariable adjustment in men, women in the highest UPF quartile group had approximately 50% greater odds of NAFLD compared to those in the lowest quartile group. These findings highlight sex-specific vulnerability, suggesting that the harmful effects of UPF consumption on liver health may be stronger in women, a critical pattern that would have been obscured without a stratified analysis. This investigation is one of the few large-scale studies in an Asian population. In addition, to our best knowledge, this study is the first to explore potential sex-specific differences in the association between UPF and NAFLD, providing evidence to inform tailored dietary guidelines and public health strategies.

Our results are consistent with previous evidence indicating that UPF consumption adversely affects metabolic health. Recent investigations have reported a positive association between high UPF intake and risk of NAFLD. In a prospective analysis of a Tianjin cohort in China (*n* = 16,000), individuals in the highest category of UPF consumption had a significantly higher risk of incident NAFLD [19]. Similarly, a recent systematic review and meta-analysis showed a significant association between UPF intake and NAFLD [20]. Moreover, a systematic review by Grinshpan et al. [21] summarized the limited and heterogeneous evidence, noting that only half of the available studies—mostly cross-sectional and conducted in Western populations—reported significant associations between UPF and NAFLD. Our findings extend the current evidence base by providing large-scale, nationally representative data from an Asian population, thereby addressing one of the key gaps identified in that review.

These associations are biologically plausible as UPFs can increase spontaneous energy intake owing to their high palatability and energy density, promoting weight gain and central adiposity, with consequent insulin resistance [22]. In addition, added sugars and refined fats may enhance hepatic de novo lipogenesis, thereby augmenting intrahepatic triglyceride accumulation [23] and contributing to NAFLD development and progression. Collectively, these mechanisms suggest that UPF-centered dietary patterns may increase the risk of hepatic fat accumulation, even among younger adults. In contrast, a cross-sectional study found no association between energy intake from UPF and fat mass [12,24], although the limited sample size of this study may have reduced the statistical power and increased the likelihood of type II errors.

To the best of our knowledge, no previous investigation has explicitly stratified the association between UPF and NAFLD according to sex, limiting direct comparisons. The current study found a positive association between UPF intake and NAFLD among females. Notably, this female-specific association contrasts with reports of related outcomes in which UPF effects appeared stronger in men. For example, Lee et al. found that the association between obesity and higher UPF consumption was stronger in men than in women [25]. Collectively, these findings suggest that the health effects of UPFs may vary by target organ and pathogenic pathways and are modulated by sex-related biological susceptibility.

Premenopausal women are relatively protected against visceral adiposity and insulin resistance through estrogenic effects; consequently, NAFLD is generally less prevalent even among women with obesity than among men [26]. Meanwhile, the risk of NAFLD among postmenopausal women is similar to or even exceeds that of men [27]. In addition, compared to men, women have higher peripheral and hepatic insulin sensitivity and show greater suppression of hepatic glucose production, conferring a metabolically favorable profile [28,29]. The hepatic de novo lipogenesis response to carbohydrate overfeeding is also lower in women than in men [30], consistent with a stronger intrinsic defense against hepatic fat accumulation. However, the energy surplus and poor nutrient composition of UPF diets can offset these protective effects. Women generally have smaller body size and lower basal metabolic rates than men [31]; therefore, a caloric excess may impose a proportionally greater storage burden. When the subcutaneous buffer capacity, supported in part by estrogen, is exceeded, fat partitioning can shift toward visceral and ectopic (hepatic) depots, potentially increasing the risk of NAFLD.

In contrast, men tend to have higher baseline propensities for visceral fat accumulation and NAFLD [26,32]; thus, the additional risk attributable to UPF may appear less pronounced on a relative scale. Collectively, these mechanisms may provide a biologically plausible explanation for the stronger association between UPF intake and NAFLD observed in women in our study.

This study leveraged a large, nationally representative sample of Korean adults from the KNHANES 2013–2021 (*n* = 24,587), with the full application of survey weights, stratification, and clustering, thereby maximizing statistical power and enhancing external validity. Beyond the overall association, sex-stratified analyses identified meaningful heterogeneity, revealing a stronger association between UPF and NAFLD in women that could have been obscured in the aggregate models. These findings provide a rationale for sex-tailored clinical and public health messaging. Finally, the evidence for the dose–response relationship was demonstrated on both categorical and continuous scales—through quartile-based logistic regression with tests for linear trend and complementary continuous modeling of %UPF in multivariable logistic regression—strengthening the robustness and biological plausibility of the findings.

This study has limitations that warrant consideration. First, its cross-sectional design precludes causal inferences. Second, the exposure is determined from a single 24 h dietary recall that may not have captured usual intake and is vulnerable to recall error. Moreover, applying NOVA categories to the Korean diet, where mixed dishes and composite recipes are common, introduces potential misclassification. Third, despite adjustments for key demographic, socioeconomic, lifestyle, and dietary factors, residual confounding factors remain. Unmeasured or imprecisely measured determinants such as genetic susceptibility, menopausal status, hormone therapy, detailed alcohol use patterns, sleep, and psychosocial stress can influence both UPF consumption and NAFLD risk. The magnitude of the association should be interpreted with caution given these limitations. Longitudinal studies with repeated dietary assessments, imaging-based endpoints, and finer control of sex-specific and genetic factors are needed.

Our findings indicate that reducing UPF intake may contribute to the prevention of NAFLD and promotion of liver health. Given the recent transition of nomenclature from non-alcoholic fatty liver disease (NAFLD) to metabolic dysfunction–associated steatotic liver disease (MASLD) [3], our results also align with the updated conceptual framework, highlighting UPF consumption as a modifiable metabolic risk factor under both terms. Clinicians should consider counseling that prioritizes eating a minimally processed diet and gradually reduces the frequency and portion size of high-contributing UPFs. Where feasible, dietitian referral, use of brief food diaries, and basic label-reading instructions can improve feasibility through patient-tailored strategies. Given the stronger association observed in women, sex-informed counseling such as focused education and follow-up for women in their midlife, a period marked by hormonal transition and fat redistribution, appears to be clinically reasonable.

Future research should use prospective cohorts with repeated dietary assessments to establish temporality and quantify dose–response relationships with greater precision. Interventional work spanning pragmatic nutrition counseling to structured UPF reduction trials should determine whether lowering UPF intake improves liver fat, ideally with imaging-based endpoints and related biomarkers. Studies should also explicitly examine effect modification by sex, menopausal status, and sex hormones and incorporate stratification by genetic susceptibility.

## 5. Conclusions

In this analysis of a nationally representative sample of Korean adults from the 2013–2021 KNHANES, higher UPF intake was associated with a higher prevalence of NAFLD. A consistent dose–response association was observed in both quartile-based logistic models and continuous analyses, with the association more pronounced in women after multivariable adjustment. Although the cross-sectional nature of the study limits its ability to establish causality, the convergence of categorical and continuous analyses, along with biological plausibility, supports the reduction in UPF consumption as a feasible approach to improve liver health. Prospective imaging-based cohort studies and intervention trials, along with analyses that consider sex and menopausal status, are needed to establish causality, quantify effect size, and inform sex-aware clinical guidance and policies.

## Figures and Tables

**Table 1:** Baseline characteristics of study population by quartiles of %UPF intake in total foods consumed.

	Quartile of %UPF Intake in Total Foods Consumed					*p*-Value
	Total	Q1	Q2	Q3	Q4	
n	24,587	6147	6146	6148	6146	
%UPF in total food intake, median (IQR)	12.85 (4.96, 26.58)	1.89 (0.91, 3.10)	7.57 (5.88, 9.39)	16.77 (13.91, 20.03)	36.80 (29.52, 47.24)	-
Age	43.66 ± 15.66	48.18 ± 16.33	46.74 ± 16.17	43.43 ± 15.60	37.75 ± 12.83	<0.001
Sex						<0.001
Men	10,419 (48.48)	2118 (39.11)	2269 (41.89)	2708 (50.16)	3324 (59.86)	
Women	14,168 (51.52)	4029 (60.89)	3877 (58.11)	3440 (49.84)	2822 (40.14)	
BMI	23.81 ± 3.69	23.59 ± 3.64	23.74 ± 3.65	23.84 ± 3.61	24.01 ± 3.75	<0.001
Education						<0.001
Elementary school or lower	3166 (9.10)	976 (12.07)	931 (10.85)	803 (9.22)	456 (5.16)	
Middle school	1862 (6.40)	539 (7.53)	539 (7.87)	459 (6.20)	325 (4.45)	
High school	8209 (38.12)	1858 (33.67)	1968 (35.96)	1986 (37.58)	2397 (43.98)	
College or higher	10,055 (46.37)	2459 (46.74)	2423 (45.32)	2552 (47.00)	2621 (46.41)	
Household income						<0.001
Low	3471 (11.40)	976 (12.89)	922 (12.18)	846 (10.94)	727 (9.97)	
Lower middle	5882 (23.32)	1434 (22.48)	1497 (23.17)	1474 (23.28)	1477 (24.15)	
Upper middle	7186 (31.00)	1750 (30.11)	1737 (29.76)	1802 (31.50)	1897 (32.28)	
High	7960 (34.29)	1964 (34.52)	1969 (34.90)	2005 (34.28)	2022 (33.60)	
Alcohol consumption						<0.001
At least once per month	13,559 (60.07)	2725 (48.79)	2962 (52.98)	3470 (59.73)	4402 (75.24)	
Less than once per month	10,552 (39.93)	3304 (51.21)	3083 (47.02)	2565 (40.27)	1600 (24.76)	
Smoking status						<0.001
Non-smokers	20,556 (80.84)	5552 (88.70)	5390 (85.91)	5113 (80.51)	4501 (70.70)	
Smokers	4031 (19.16)	595 (11.30)	756 (14.09)	1035 (19.49)	1645 (29.30)	
Physical activity						<0.001
Low	11,005 (49.91)	2821 (51.72)	2852 (51.16)	2777 (50.59)	2555 (46.82)	
High	9768 (50.09)	2379 (48.28)	2409 (48.84)	2398 (49.41)	2582 (53.18)	
Total energy intake, kcal/day	2154.04 ± 961.75	1940.07 ± 863.30	2052.91 ± 848.71	2179.13 ± 938.42	2385.35 ± 1049.60	<0.001
Men, n	10,419	2118	2269	2708	3324	
%UPF in total food intake, median (IQR)	16.21 (6.44, 30.85)	2.02 (0.96, 3.23)	7.75 (5.91, 9.46)	17.03 (14.08, 20.24)	37.35 (29.82, 48.03)	-
Age	43.14 ± 14.48	48.52 ± 15.73	47.16 ± 15.15	43.11 ± 14.27	38.05 ± 12.02	<0.001
BMI	24.61 ± 3.29	24.47 ± 3.22	24.64 ± 3.32	24.54 ± 3.15	24.73 ± 3.36	0.058
Education						<0.001
Elementary school or lower	1016 (6.34)	278 (9.41)	265 (7.39)	269 (6.68)	204 (3.89)	
Middle school	772 (5.88)	184 (6.92)	219 (8.22)	204 (5.80)	165 (4.02)	
High school	3597 (39.64)	621 (33.79)	736 (36.02)	914 (39.03)	1326 (45.27)	
College or higher	4402 (48.13)	896 (49.89)	946 (48.37)	1148 (48.49)	1412 (46.82)	
Household income						0.128
Low	1421 (10.55)	341 (12.17)	339 (11.54)	338 (9.58)	403 (9.85)	
Lower middle	2438 (22.62)	509 (22.36)	550 (22.39)	642 (22.45)	737 (23.01)	
Upper middle	3087 (31.68)	584 (29.46)	650 (30.53)	822 (32.76)	1031 (32.71)	
High	3440 (35.15)	677 (36.01)	723 (35.54)	898 (35.21)	1141 (34.43)	
Alcohol consumption						<0.001
At least once per month	7286 (72.68)	1292 (64.36)	1472 (67.18)	1894 (70.87)	2628 (81.56)	
Less than once per month	2890 (27.32)	776 (35.64)	760 (32.82)	758 (29.13)	596 (18.44)	
Smoking status						<0.001
Non-smokers	7040 (66.05)	1635 (75.64)	1644 (71.77)	1826 (66.11)	1935 (57.73)	
Smokers	3379 (33.95)	483 (24.36)	625 (28.23)	882 (33.89)	1389 (42.27)	
Physical activity						0.034
Low	4291 (46.14)	884 (47.39)	995 (47.74)	1123 (47.30)	1289 (43.68)	
High *	4431 (53.86)	8883 (52.61)	950 (52.26)	1128 (52.70)	1470 (56.32)	
Total energy intake, kcal/day	2538.94 ± 972.17	2282.26 ± 916.31	2435.22 ± 842.66	2534.70 ± 945.45	2735.15 ± 1029.32	<0.001
Women, n	14,168	4029	3877	3440	2822	
%UPF in total food intake, median (IQR)	10.26 (3.97, 22.14)	1.80 (0.87, 3.00)	7.41 (5.86, 9.29)	16.46 (13.76, 19.79)	35.70 (29.01, 46.26)	-
Age	44.116 ± 16.72	47.966 ± 16.65	46.44 ± 16.87	43.75 ± 16.91	37.30 ± 14.00	<0.001
BMI	23.05 ± 3.90	23.02 ± 3.78	23.10 ± 3.74	23.13 ± 3.93	22.94 ± 4.10	0.614
Education						<0.001
Elementary school or lower	2150 (11.65)	698 (13.73)	666 (13.35)	534 (11.73)	252 (7.03)	
Middle school	1090 (6.89)	355 (7.91)	320 (7.62)	255 (6.61)	160 (5.07)	
High school	4612 (36.72)	1237 (33.60)	1232 (35.92)	1072 (36.14)	1071 (42.10)	
College or higher	5653 (44.74)	1563 (44.77)	1477 (43.11)	1404 (45.52)	1209 (45.80)	
Household income						0.009
Low	2050 (12.20)	635 (13.36)	583 (12.63)	508 (12.31)	324 (10.14)	
Lower middle	3444 923.98)	925 (22.55)	947 (23.74)	832 (24.11)	740 (25.85)	
Upper middle	4099 (30.35)	1166 (30.53)	1087 (29.19)	980 (30.24)	866 (31.64)	
High	4520 (33.47)	1287 (33.57)	1245 (34.43)	1107 (33.34)	881 (32.36)	
Alcohol consumption						<0.001
At least once per month	6273 (48.28)	1433 (38.85)	1490 (42.75)	1576 (48.59)	1774 (65.91)	
Less than once per month	7662 (51.72)	2528 (61.15)	2323 (57.25)	1807 (51.41)	1004 (34.09)	
Smoking status						<0.001
Non-smokers	13,516 (94.75)	3917 (97.09)	3746 (96.11)	3287 (95.01)	2566 (90.04)	
Smokers	652 (5.25)	112 (2.91)	131 (3.90)	153 (4.99)	256 (9.96)	
Physical activity						0.246
Low	6714 (53.40)	1937 (54.41)	1857 (53.62)	1654 (53.83)	1266 (51.43)	
High	5337 (46.60)	1496 (45.59)	1459 (46.38)	1270 (46.17)	1112 (48.57)	
Total energy intake, kcal/day	1791.90 ± 753.35	1720.30 ± 716.02	1777.29 ± 711.02	1821.27 ± 740.58	1863.67 ± 827.36	<0.001

* High physical activity is defined as 150 min/week of moderate-intensity aerobic physical activity, 75 min/week of vigorous-intensity aerobic physical activity, or 150 min/week of a combination of moderate- and vigorous-intensity aerobic activities. Otherwise, physical activity is low. Values are presented as the mean ± standard deviation or number (%) by descriptive or frequency analysis. The *p*-values are calculated using analysis of variance or Pearson’s chi-squared test.

**Table 2:** Risk of NAFLD associated with UPF intake.

	Quartile of %Total Food Intake from UPF				*p* for Trend
	Q1	Q2	Q3	Q4	
All	6147	6146	6148	6146	
No. of cases (%)	1122 (19.12)	1260 (21.44)	1302 (22.39)	1440 (24.11)	
Unadjusted	Ref	**1.15 (1.04, 1.28)**	**1.22 (1.10, 1.36)**	**1.34 (1.21, 1.49)**	**<0.001**
Age, sex adjusted	Ref	**1.13 (1.02, 1.26)**	**1.13 (1.02, 1.26)**	**1.16 (1.04, 1.30)**	**0.012**
Multivariable adjusted **	Ref	**1.17 (1.04, 1.31)**	**1.18 (1.05, 1.33)**	**1.24 (1.10, 1.41)**	**0.001**
Men	2118	2269	2708	3324	
No. of cases (%)	471 (25.06)	593 (28.33)	685 (27.46)	934 (28.83)	
Unadjusted	Ref	**1.18 (1.01, 1.39)**	**1.13 (0.97, 1.32)**	**1.21 (1.05, 1.40)**	**0.032**
Age adjusted	Ref	1.16 (0.98, 1.36)	1.02 (0.87, 1.19)	0.99 (0.85, 1.15)	0.383
Multivariable adjusted *	Ref	1.18 (0.99, 1.42)	1.08 (0.91, 1.29)	1.06 (0.89, 1.26)	0.943
Women	4029	3877	3440	2822	
No. of cases (%)	651 (15.31)	667 (16.47)	617 (17.29)	506 (17.09)	
Unadjusted	Ref	1.09 (0.95, 1.25)	1.16 (1.00, 1.34)	1.14 (0.98, 1.32)	0.054
Age adjusted	Ref	**1.13 (0.98, 1.29)**	**1.26 (1.09, 1.46)**	**1.44 (1.23, 1.68)**	**<0.001**
Multivariable adjusted *	Ref	1.15 (0.99, 1.34)	**1.29 (1.09, 1.51)**	**1.52 (1.28, 1.81)**	**<0.001**

** Adjusted for age, sex, education level, household income, smoking status, alcohol consumption, physical activity, and total energy intake. * Adjusted for age, education level, household income, smoking status, alcohol consumption, physical activity, and total energy intake. The *p* for trend is calculated using logistic regression analysis. Bold values indicate statistical significance (*p* < 0.05).

## Data Availability

The data used in this study are publicly available and can be freely downloaded from the KNHANES website (https://knhanes.kdca.go.kr/ (accessed on 1 August 2025)).

## Abbreviations

The following abbreviations are used in this manuscript:

BMI	body mass index
CIs	confidence intervals
HSI	hepatic steatosis index
KNHANES	Korea National Health and Nutrition Examination Survey
NAFLD	non-alcoholic fatty liver disease
ORs	odds ratios
UPFs	ultra-processed foods
